# *Ficus microcarpa* Bonsai “Tiger bark” Parasitized by the Root-Knot Nematode *Meloidogyne javanica* and the Spiral Nematode *Helicotylenchus dihystera*, a New Plant Host Record for Both Species

**DOI:** 10.3390/plants9091085

**Published:** 2020-08-24

**Authors:** Duarte Santos, Isabel Abrantes, Carla Maleita

**Affiliations:** 1 CFE, Department of Life Sciences, University of Coimbra, Calçada Martim de Freitas, 3000 456 Coimbra, Portugal; duartenema@outlook.pt (D.S.); isabel.abrantes@uc.pt (I.A.); 2 CIEPQPF, Department of Chemical Engineering, University of Coimbra, Rua Sílvio Lima, Pólo II–Pinhal de Marrocos, 3030-790 Coimbra, Portugal

**Keywords:** 28S ribosomal DNA, mitochondrial DNA region, pest interception, plant-parasitic nematodes, SCAR-PCR

## Abstract

In December 2017, a *Ficus microcarpa* “Tiger bark” bonsai tree was acquired in a shopping center in Coimbra, Portugal, without symptoms in the leaves, but showing small atypical galls of infection caused by root-knot nematodes (RKN), *Meloidogyne* spp. The soil nematode community was assessed and four Tylenchida genera were detected: *Helicotylenchus* (94.02%), *Tylenchus* s.l. (4.35%), *Tylenchorynchus* s.l. (1.09%) and *Meloidogyne* (0.54%). The RKN *M. javanica* was identified through analysis of esterase isoenzyme phenotype (J3), PCR-RFLP of mitochondrial DNA region between COII and 16S rRNA genes and SCAR-PCR. The *Helicotylenchus* species was identified on the basis of female morphology that showed the body being spirally curved, with up to two turns after relation with gentle heat, a key feature of *H. dihystera*, and molecular characterization, using the D2D3 expansion region of the 28S rDNA, which revealed a similarity of 99.99% with available sequences of the common spiral nematode *H. dihystera*. To our knowledge, *M. javanica* and *H. dihystera* are reported for the first time as parasitizing *F. microcarpa*. Our findings reveal that more inspections are required to detect these and other plant-parasitic nematodes, mainly with quarantine status, to prevent their spread if found.

## 1. Introduction

The globalization era opens up new trade routes and increases the volume and complexity of cross-border transactions of goods. The plant sector (plant products, germplasm, grafts and live plants) has been part of the general trend in increased trade. This exchange of species between distant geographical regions of the globe creates new pathways for the introduction of alien plant pests and diseases [1,2].

The introduction of a non-native organism in a new environment produces unpredictable effects. A species may have low impact in its native range, but much greater impact when introduced to new areas, putting native biodiversity and local production systems at risk [1,3,4]. For instance, the introduction of the alien pinewood nematode *Bursaphelenchus xylophilus* in Portugal has caused huge environmental and economic losses in Portuguese pine forests, while in North America, where this nematode is native, it does not cause significant mortality to native conifers [5,6,7]. These problems are expected to be intensified in the future as climate change is predicted to facilitate the further spread of these species, since many of these new pathogens are of tropical and subtropical origins [8,9]. Recently, the tropical root knot nematode (RKN) *Meloidogyne luci* (Alert List of the European and Mediterranean Plant Protection Organization—EPPO) and *M. enterolobii* (A2 List of Pests EPPO) were detected in Portugal. *Meloidogyne luci* was found to be associated with potato (*Solanum tuberosum*) and tomato (*S. lycopersicum*), the ornamental plant *Cordyline australis*, and the weed *Oxalis corniculata*, whereas *M. enterolobii* was detected in the ornamental plants *Cereus hildmannianus*, *Lampranthus* sp., *Physalis peruviana* and *Callistemon* sp. Taking into account its aggressiveness and distribution, there is a high probability of spread in the Mediterranean region and also in Europe, becoming a potential threat to the agricultural economy [10,11].

The European Commission has proposed an import ban on 35 genera of plants for planting, other than seeds, in vitro material and natural or artificially dwarfed woody plants for planting from countries outside the European Union (EU). *Ficus carica*, common fig, is the only species of the genus *Ficus* included on the list. The ban was put into effect in December 2019 and aims to reduce the probability of the introduction of harmful organisms in the EU [12]. During a survey (3 years) in the Netherlands, around 20% of samples of imported plants for planting and ornamentals from 21 countries showed quarantine nematodes and 11% other important nematodes [13].

*Ficus* constitutes one of the largest genera of flowering plants (Angiosperms) that, according to the plant list version 1.1 (http://www.theplantlist.org), has 919 accepted species, being primarily found in tropical and subtropical environments throughout the world [14]. During the past few decades, plants from this genus have become quite popular as indoor house plants.

Despite the diversity of *Ficus* species, the research regarding plant-parasitic nematodes (PPN) has been focussed on the edible fig tree *F. carica* native to western Asia and introduced in the Mediterranean region. A number of PPN species have been reported as parasitizing fig trees in many countries, the most prevalent belonging to the genera *Helicotylenchus* (spiral nematodes), *Heterodera* (cyst nematodes), *Meloidogyne* (root-knot nematodes), *Paratylenchus* (pin nematodes), *Pratylenchus* (root lesion nematodes, RLN) and *Xiphinema* (dagger nematodes) [15,16,17,18,19,20,21,22,23,24]. Of these, the most common are the RKN species *M. arenaria*, *M. hapla*, *M. hispanica*, *M. incognita* and *M. javanica*, economically important species that directly target plant roots and prevent water and nutrient uptake, resulting in growth or even plant death in extreme cases, and the species *Heterodera fici*, a worldwide parasite of ornamental and cultivated *Ficus* species [24,25].

Concerning *Ficus* bonsai, besides *F. carica*, other species, such as *F. benghalensis*, *F. macrophylla*, *F. microcarpa*, *F. retusa* and *F. rubiginosa*, have been considered suitable for bonsai plants, but records of PPN on them are few and scarce. Some bonsai plants with nematode infections have been intercepted in Europe and other parts of the world [13,26,27].

The species *F. microcarpa*, the Indian laurel tree, sometimes confused with *F. nitida* and *F. retusa*, is widely distributed as an ornamental plant either outdoors or indoors and is known for its pharmacological properties: antioxidant, antibacterial, anticancer, anti-diabetic, anti-diarrhoeal, anti-inflammatory, anti-asthmatic, hepatoprotective and hypolipidemic [28]. This *Ficus* species is a host of many pests, including the Cuban laurel thrips (*Gynaikothrips ficorum*), the Ficus leaf-rolling psyllid (*Trioza brevigenae*), and the Ficus whitefly (*Singhiella simplex*), among others [29]. The PPN found associated with *F. microcarpa* include the genera *Helicotylenchus*, *Meloidogyne*, *Pratylenchus*, *Tylenchorynchus* and *Xiphinema*, and the RKN *M. enterolobii* species have been intercepted in bonsais or plants for planting imported from China or Egypt [27].

**Figure 1:**
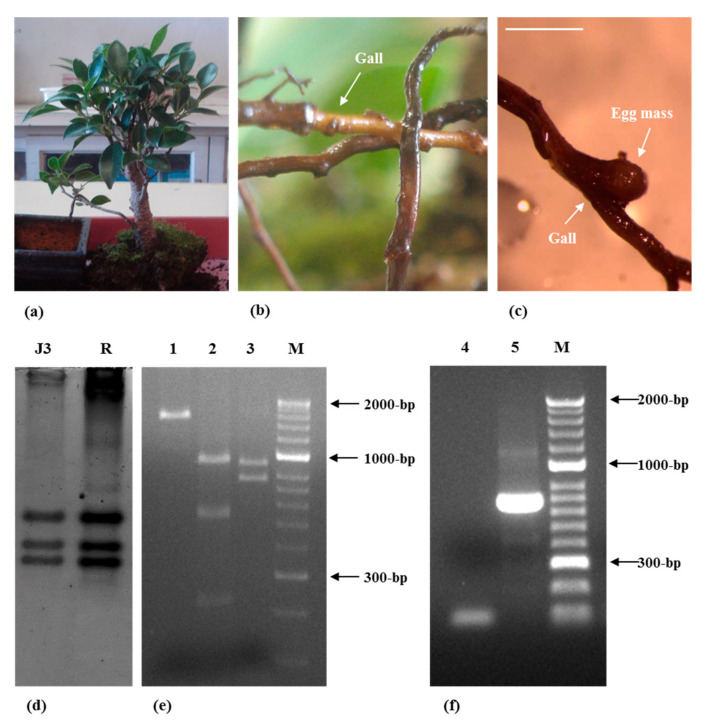
*Meloidogyne javanica* parasitizing *Ficus microcarpa*. (**a**) *F. microcarpa*. (**b**,**c**) *F. microcarpa* infected roots. (**d**) Polyacrylamide gel stained for esterase activity. J3, *M. javanica* (*F. microcarpa* isolate); R, *M. javanica* (reference isolate). (**e**) *Hinf*I (1), *Alu*I (2) and *Dra*III (3) digestion patterns of the approximately 1800-bp amplification products from *M. javanica*, using C2F3 and MRH106 primers. M, DNA marker (HyperLadder II; Bioline). (**f**) DNA amplification product using Fjav and Rjav primers. 4, Negative control; 5, *M. javanica*; M, DNA marker (HyperLadder II; Bioline). Scale bar: 1 mm.

The aims of the present study were to find the nematode diversity associated to *F. mircrocarpa* bonsai plant, to characterize/identify the RKN, *Meloidogyne* sp., and the spiral nematode, *Helicotylenchus* sp., parasitizing *F. microcarpa* (Figure 1a) and to enlarge the knowledge on the phytoparasitic nematodes of this *Ficus* species.

## 2. Results and Discussion

**Figure 2:**
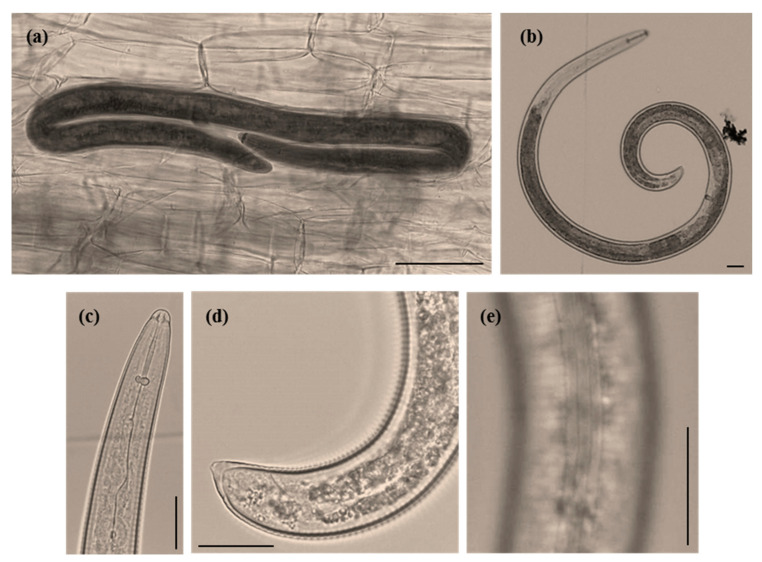
*Helicotylenchus dihystera* (females) light microscope photographs. (**a**) Infected *Ficus microcarpa* root. (**b**) Whole specimen. (**c**) Anterior region in lateral view. (**d**) Posterior region in lateral view. (**e**) Lateral field with four lateral lines. Scale bars: 20 µm (**a**–**d**), and 50 µm (**e**).

*Meloidogyne* females plus egg masses (Figure 1b,c) and *Helicotylenchus* specimens (Figure 2a) were detected in fresh and stained roots. Although *Helicotylenchus* spp. are classified as ectoparasites or semi-endoparasites, they can penetrate the roots and were already found completely embedded in the cortical tissue of the root system of sycamore (*Platanus occidentalis*) [30]. The galls were small and hard, mainly in woody roots (Figure 1b,c), which is common in woody perennial plants. The PPN detected in the soil sample (130 g) of *F. microcarpa* bonsai belonged to four genera: *Helicotylenchus* (94.02%), *Tylenchus* s.l. (4.35%), *Tylenchorynchus* s.l. (1.09%) and *Meloidogyne* J2 (0.54%). The spiral nematode *Helicotylenchus* was the most prevalent PPN, detected in very high numbers, with approximately 3000 nematodes.

For the RKN isolate, the biochemical characterization resulted in three bands of esterase (relative mobility %: 0.38; 0.45; 0.49), which is the characteristic phenotype exhibited by *M. javanica* (J3) isolates (Figure 1d). The mtDNA *COII* and 16S rRNA genes region amplified with the primer set C2F3/MRH106 yielded a single fragment of 1800 bp. When the amplified product was digested with the restriction enzyme *Hinf*I, no digestion occurred. *Alu*I and *Dra*III generated three fragments of approximately 1000, 580, and 240 bp and two fragments of approximately 1000 and 800 bp, which is in accordance with other results for this species [31] (Figure 1e). Additionally, molecular characterization of the RKN species with the species-specific primers Fjav and Rjav produced a fragment size of 600 bp, as expected, thus confirming the presence of *M. javanica* (Figure 1f).

This RKN species is known to parasitize *F. carica* and is one of the most widely distributed species and the second highest in economic importance after *M. incognita* [22,23,32]. *Meloidogyne javanica* was first reported from Portugal on potato in Azores [33]. Since then, it has been found on several economically important crops, including *Humulus lupulus*, *Musa* sp., *Phaseolus vulgaris*, *Prunus persica*, *S. lycopersicum* and *S. tuberosum*, ornamental plants, such as *Cordyline australis* and *Dianthus plumarius*, as well as many other dicots [11,34,35,36,37,38,39,40].

**Figure 3:**
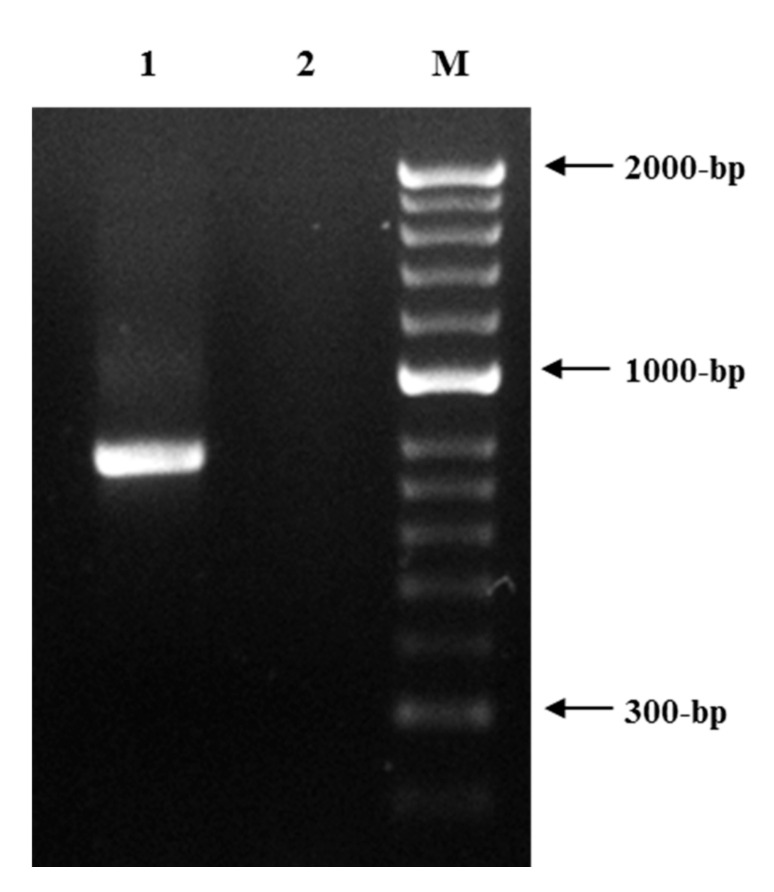
DNA amplification product obtained from *Helicotylenchus dihystera* isolate identified on *Ficus microcarpa* to the D2D3 expansion region of the 28S rDNA gene (1). 2, negative control. M, DNA marker (HyperLadder II; Bioline).

**Figure 4:**
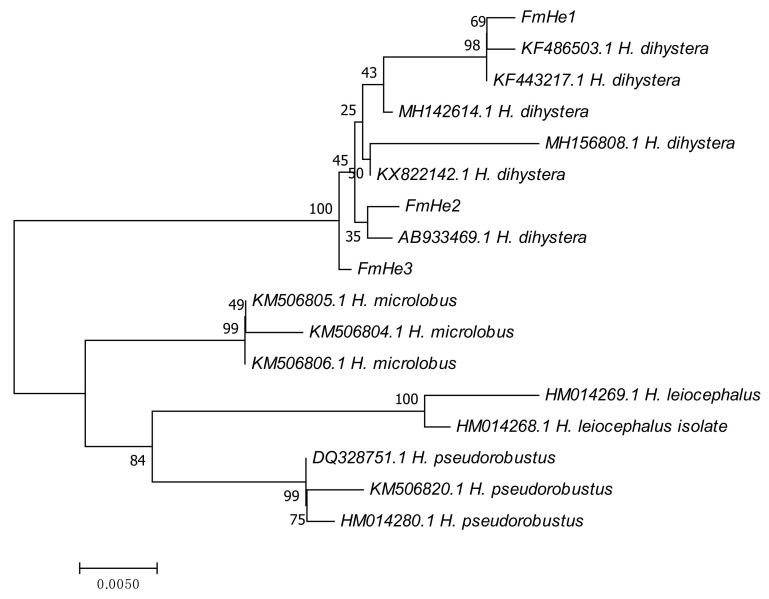
Neighbor-joining tree based on analysis of alignment of D2D3 expansion region of the 28S rDNA gene sequences of the *Helicotylenchus dihystera* isolate identified on *Ficus microcarpa* (FmHe) with available sequences of close *Helicotylenchus* spp. (*H. leiocephalus*, *H. microlobus* and *H. pseudorobustus*) [41]. The percentage of replicate trees in which the associated *Helicotylenchus* spp. clustered together in the bootstrap test (1000 replicates) is shown next to the branches. Evolutionary distances were computed using the maximum composite likelihood method and all positions containing gaps and missing data were eliminated.

*Helicotylenchus* females were spirally curved, with up to two turns after relation with gentle heat, a key feature of *H. dihystera* (Figure 2b), the tail dorsally convex-conoid to a narrow terminus with a slight projection (Figure 2d) and lateral field with four non-areolated incisures (Figure 2e). Males were not found [41]. Amplification of the D2D3 expansion region of the 28S rDNA gene resulted in a product of ca. 750 bp (Figure 3). Sequences (744 bp) were submitted to the GenBank database with accession numbers MT277384, MT277385 and MT277386. The three sequences of the *Helicotylenchus* sp. from *Ficus microcarpa* (Fm) were compared and nine nucleotide changes at positions 57, 77, 98, 189, 537, 541, 546, 548 and 572 in alignment were identified. The comparison of this region with the *Helicotylenchus* sequences available in the GenBank database revealed a similarity of 99.99% with *H. dihystera.* Phylogenetic analysis from the alignment of Fm *Helicotylenchus* 28S rDNA sequences with available sequences of similar *Helicotylenchus* spp. [41] revealed that this isolate and all listed *H. dihystera* sequences appeared together in a well-separated clade with 100% bootstrap support (Figure 4)*,* confirming the morphological identification. Considering a common start and end point to *Helicotylenchus* spp. (560 bp), the Fm sequences differed in several positions from at least one *H. dihystera* sequences included in the analysis; however, only three (4, 493 and 519) position changes were completely distinct from *H. dihystera* sequences (Figure S1). Fm *H. dihystera* sequences had divergences ranging from 0.2 to 2.2% when compared with *H. dihystera* sequences and 3.7 to 6.5% to the other *Helicotylenchus* species (Table S1).

Although *H. dihystera* is considered as a polyphagous species with a wide distribution, reports on its pathogenicity are very few, and it is rarely recognized as an economically important PPN [30,42,43,44], even when high population densities are found. This is the first report of *H. dihystera* infecting *F. microcarpa;* however, it has been associated with *F. benjamina, F. carica*, *F. elastica, F. formosana* and *F. retusa* [21,26,44,45,46,47]. In Portugal, *H. dihystera* was reported as being associated with *Begonia* sp., *Colocasia esculenta*, *Cactus* sp., *Mentha* sp., *Musa* sp., *Pelargonium* sp., Polygonaceae, beans, maize and tomato [45,48,49,50].

Although no specific quarantine measures are being implemented against *M. javanica* or *H. dihystera*, preventive measures are particularly important to decrease the risk of spread into a region where they do not exist. Once nematodes are established in the soil, their eradication is very difficult. Thus, the use and transportation of clean, healthy, nematode-free planting material is a prerequisite for limiting the spread of nematodes. Plant parts liable to carrying PPN in trade/transport can be bulbs/tubers/corms/rhizomes, growing medium accompanying plants, roots and micropropagated plants. During routine inspections, the detection of nematode infections can be easily overlooked or misdiagnosed, as low to moderate populations of nematodes may cause no visible aboveground symptoms, making it harder to diagnose them [51,52]. Furthermore, above-ground symptoms are non-specific and usually involve stunting, lack of vigour, leaf nutritional deficiencies and temporary wilting in periods of water stress and high temperatures. The examination of roots can reveal the presence of galls that are specific symptoms associated with the occurrence of *Meloidogyne* spp., but the symptoms caused by *Helicotylenchus* spp., when present, can be confused with the damage associated with poor nutrition or injury caused by pathogens that attack the root system (other nematodes, bacteria, fungi and/or virus).

To our knowledge, *M. javanica* and *H. dihystera* are here reported for the first time as parasitizing *F. microcarpa*. Although both PPN species are common and widely distributed, our findings emphasize the importance of inspections by governmental authorities to find out whether imported material is free of PPN. If plants are grown in infested soil and then commercialized, it increases the probability of PPN dissemination to new regions and/or other suitable hosts with potential impact on economically important crops.

## 3. Materials and Methods

In December 2017, a *F. microcarpa* “Tiger bark” bonsai tree with a phytosanitary certificate was acquired by the first author in a shopping center in Coimbra, Portugal, showing small galls in the protruding roots, which aroused our attention, but without symptoms in the leaves (Figure 1a,b). Consequently, a few roots and a soil sample of 130 g were collected from the pot. Roots were observed directly and stained with acid fuchsin to detect nematode-infected plant tissues [53]. Nematodes were extracted from the soil, according to the Tray Method [54], followed by microscopic examination of nematode diversity and the genera identified and quantified.

### 3.1. Root Knot Nematode Characterization/Identification

Egg masses from *F. microcarpa* galled roots were propagated on tomato, *Solanum lycopersicum*, cv. Coração-de-Boi, in a growth chamber. After two months, the infected tomato roots were gently rinsed with tap water and 5 young egg-laying females were, individually and randomly, handpicked with their respective egg masses to glass blocks with NaCl 0.9% to obtain pure cultures. Individual young egg-laying females were characterised biochemically by electrophoretic analysis of esterases. Esterase electrophoresis was performed using polyacrylamide gels following the methodology described by Pais and Abrantes [55]. The individual females were transferred to micro-haematocrit tubes containing 5 µL of extraction buffer (20% sucrose and 1% Triton X-100), macerated and stored at −20 °C. Before electrophoresis, the samples were centrifuged at 8905 *g*, at −5 °C for 15 min. Electrophoresis was performed at 6 mA/gel during the first 15 min and then at 20 mA/gel for about 45 min using the Mini-Protean Tetra System (Bio-Rad Laboratories, Hercules, CA, USA). The gels were stained for esterase activity with the substrate α-naphthyl acetate, in the dark at 37 °C. Protein extract from five females of *M. javanica* was included in each gel as a reference. A pure culture (designated as Fm) of RKN was established by inoculating the 5 individual egg masses onto tomato to obtain a sufficient number of second-stage juveniles for molecular characterization. Electrophoretic analysis of esterases was repeated after two months to confirm the biochemical identification and the relative movement of each band calculated taking as reference the buffer front (Relative mobility, Rm%).

Biochemical identification was further confirmed by PCR-RFLP of mtDNA region between COII and 16S rRNA genes with C2F3 and MRH106 primers, and by SCAR-PCR with the species-specific primers Fjav and Rjav, using a pellet of second-stage juveniles (J2) obtained from egg masses of the pure isolate [31,56]. Briefly, for mtDNA region amplification, each PCR contained 1X PCR buffer, 1.8 mM MgCl_2_, 0.2 mM dNTPs, 0.2 μM of each primer, 2.5 U Taq DNA polymerase (Bioline), and 50 ng DNA. Amplification was conducted using the following conditions: initial denaturation at 94 °C for 4 min, followed by 40 cycles of 94 °C for 30 s, 60 °C for 30 s, and 72 °C for 60 s, and a final extension for 10 min at 72 °C. After amplification, the PCR product was digested separately with 5 U *Hinf*I, *Alu*I and *Dra*III. For SCAR-PCR, the PCR reactions were the same as for the mtDNA region, except the primers (0.3 μM of each primer) and the amplification conditions (35 cycles of denaturation at 94 °C for 30 s, annealing at 52 °C for 30 s and extension at 72 °C for 1 min).

### 3.2. Spiral Nematode Characterization/Identification

*Helicotylenchus* specimens from soil and roots were propagated on the same tomato cultivar, in a growth chamber. Two/three months after inoculation, with approximately 3000 specimens, nematodes were extracted from roots/soil, according to the generalist Tray Method [54] and used to *Helicotylenchus* species characterization/identification and isolate maintenance, respectively. The characterization and identification of the *Helicotylenchus* species was based on the morphological characters of 10 females (body shape after relaxed with gentle heat and number of incisures) and ribosomal DNA (rDNA) sequencing.

DNA was extracted and purified from 20 spiral nematodes, extracted from tomato roots, using the DNeasy Blood and Tissue kit (QIAGEN, Valencia, CA, USA), according to the manufacturer’s instructions, and the D2D3 expansion region of the 28S rDNA gene was amplified using D2A (5′-ACA AGT ACC GTG AGG GAA AGT TG-3′) and D3B (5′-TCG GAA GGA ACC AGC TAC TA-3′) primers [57]. The PCR products were analysed on 1% agarose gel stained with GreenSafe (Nzytech), purified from the gel with the MiniElute Gel Extraction kit (QIAGEN, Valencia, CA, USA), quantified using the NanoDrop 2000C spectrophotometer (Thermo Scientific), cloned and sequenced. Sequences were compared with available close *Helicotylenchus* spp. sequences in GenBank [41]. Sequences were aligned using CLUSTALW multiple alignment in BIOEDIT software [58]. The evolutionary history was inferred using the neighbor-joining (NJ) and maximum likelihood (ML) methods in MEGA 7, as described in Santos et al. [10,59].

## Supplementary Materials

The following materials are available online at https://www.mdpi.com/2223-7747/9/9/1085/s1, Figure S1: Multiple sequence alignment of Fm *Helicotylenchus* (FmHe1, FmHe2 and FmHe3) and available close *Helicotylenchus* spp. (*H. dihystera*—AB933469.1, MH156808.1, MH142614.1, KX822142.1, KF486503.1, KF443217.1; *H. pseudorobustus*—KM506820.1, HM014280.1, DQ328751.1; *H. microlobus*—KM506806.1, KM506805.1, KM506804.1; *H. leiocephalus*—HM014269.1, HM014268.1) sequences of D2D3 expansion region of the 28S rDNA gene (560 bp), Table S1: Pairwise sequence divergences between Fm *Helicotylenchus* and available close *Helicotylenchus* spp. sequences in GenBank of D2D3 expansion region of the 28S rDNA gene using MEGA7.
