# Supplementary material for: Ficus microcarpa Bonsai “Tiger bark” Parasitized by the Root-Knot Nematode Meloidogyne javanica and the Spiral Nematode Helicotylenchus dihystera, a New Plant Host Record for Both Species
Source: Plants (Basel). 2020 Aug 24;9(9):1085. doi: 10.3390/plants9091085 (PMC7569956; doi:10.3390/plants9091085)
Supplement: Supplementary file 1 [file plants-09-01085-s001.pdf]

Table S1. Pairwise sequence divergences between Fm *Helicotylenchus* and available close *Helicotylenchus* spp. sequences in GenBank of D2D3 expansion region of the 28S rDNA gene using MEGA7 [55] [1].

	**(1)**	**(2)**	**(3)**	**(4)**	**(5)**	**(6)**	**(7)**	**(8)**	**(9)**	**(10)**	**(11)**	**(12)**	**(13)**	**(14)**	**(15)**	**(16)**
FmHe1 (1)	…	…	…	…	…	…	…	…	…	…	…	…	…	…	…	…
FmHe2 (2)	0.014	…	…	…	…	…	…	…	…	…	…	…	…	…	…	…
FmHe3 (3)	0.013	0.005	…	…	…	…	…	…	…	…	…	…	…	…	…	…
AB933469.1 *H. dihystera* (4)	0.011	0.004	0.005	…	…	…	…	…	…	…	…	…	…	…	…	…
MH156808.1 *H. dihystera* (5)	0.022	0.014	0.013	0.014	…	…	…	…	…	…	…	…	…	…	…	…
MH142614.1 *H. dihystera* (6)	0.009	0.005	0.004	0.005	0.013	…	…	…	…	…	…	…	…	…	…	…
KX822142.1 *H. dihystera* (7)	0.011	0.004	0.002	0.004	0.011	0.002	…	…	…	…	…	…	…	…	…	…
KF486503.1 *H. dihyster*a (8)	0.004	0.014	0.013	0.011	0.022	0.009	0.011	…	…	…	…	…	…	…	…	…
KF443217.1 *H. dihystera* (9)	0.002	0.013	0.011	0.009	0.020	0.007	0.009	0.002	…	…	…	…	…	…	…	…
KM506820.1 *H. pseudorobustus* (10)	0.054	0.046	0.044	0.046	0.058	0.048	0.046	0.054	0.052	…	…	…	…	…	…	…
HM014280.1 *H. pseudorobustus* (11)	0.052	0.044	0.042	0.044	0.056	0.046	0.044	0.052	0.050	0.005	…	…	…	…	…	…
DQ328751.1 *H. pseudorobustus* (12)	0.050	0.042	0.040	0.042	0.054	0.044	0.042	0.050	0.048	0.004	0.002	…	…	…	…	…
KM506806.1 *H. microlobus* (13)	0.046	0.039	0.037	0.039	0.050	0.040	0.039	0.046	0.044	0.029	0.027	0.025	…	…	…	…
KM506805.1 *H. microlobus* (14)	0.046	0.039	0.037	0.039	0.050	0.040	0.039	0.046	0.044	0.029	0.027	0.025	0.000	…	…	…
KM506804.1 *H. microlobus* (15)	0.050	0.042	0.040	0.042	0.054	0.044	0.042	0.050	0.048	0.033	0.031	0.029	0.004	0.004	…	…
HM014269.1 *H. leiocephalus* (16)	0.065	0.058	0.056	0.058	0.069	0.060	0.058	0.065	0.063	0.039	0.037	0.035	0.039	0.039	0.042	…
HM014268.1 *H. leiocephalus* (17)	0.060	0.052	0.050	0.052	0.063	0.054	0.052	0.060	0.058	0.033	0.031	0.029	0.033	0.033	0.037	0.009

[1] Analyses were conducted using the Jukes-Cantor model and all positions containing gaps and missing data were eliminated.

```
                       10         20         30         40         50
FmHe1       CGGTCAGAGC TGGCGTATCT GGCTTGCATT CAGCCGCTGC -GCGTTGGGC
FmHe2       CGGACAGAGC TGGCGTATCT GGCCTGCATT CAGCCGCTGC -GCGTCGGGC
FmHe3       CGGACAGAGC TGGCGTATCT GGCCTGCATT CAGCCGCTGC -GCGTCGGGC
AB933469.1  CGGACAGAGC TGGCGTATCT GGCCTGCATT CAGCCGCTGC -GCGTCGGGC
MH156808.1  CGGACAGAGC TGGCGTATCT GGCCTGCATT CAGCCGCTGC -GCGTCGGGC
MH142614.1  CGGACAGAGC TGGCGTATCT GGCTTGCATT CAGCCGCTGC -GCGTCGGGC
KX822142.1  CGGACAGAGC TGGCGTATCT GGCCTGCATT CAGCCGCTGC -GCGTCGGGC
KF486503.1  CGGACAGAGC TGGCGTATCT GGCTTGCATT CAGCCGCTGC -GCGTTGGGC
KF443217.1  CGGACAGAGC TGGCGTATCT GGCTTGCATT CAGCCGCTGC -GCGTTGGGC
KM506820.1  CGGACAGAGC TGGCGTATCT GGCCTGCATT CAGCCGCTGC -GCGTCGGGC
HM014280.1  CGGACAGAGC TGGCGTATCT GGCCTGCATT CAGCCGCTGC -GCGTCGGGC
DQ328751.1  CGGACAGAGC TGGCGTATCT GGCCTGCATT CAGCCGCTGC -GCGTCGGGC
KM506806.1  CGGACAGAGC TGGCGTATCT GGCCTGCATT CAGCCGCCGC -GCGTCGGGC
KM506805.1  CGGACAGAGC TGGCGTATCT GGCCTGCATT CAGCCGCCGC -GCGTCGGGC
KM506804.1  CGGACAGAGC TGGCGTATCT GGCCTGCATT CAGCCGCCGC -GCGTCGGGC
HM014269.1  CGAACAGAGC TGGCGTATCT GGCCTGCATT CAGCCGCCGC -ACGTCGGGC
HM014268.1  CGGACAGAGC TGGCGTATCT GGCCTGCATT CAGCCGCCGT TACGTCGGGC

                       60         70         80         90        100
FmHe1       CCCGGTTTGC CGCACTCCAG ATTGGGAAGG TGGCTGGGCT GCTGGCGTGC
FmHe2       CCCGGTTTGC CGCACTCCAG ATTGGGAAGG TGGCTGGGCT GCTGGCGTGC
FmHe3       CCCGGTTTGC CGCACTCCAG ATTGGGAAGG TGGCTGGGCT GCTGGCGTGC
AB933469.1  CCCGGTTTGC CGCACTCCAG ATTGGGAAGG TGGCTGGGCT GCTGGCGTGC
MH156808.1  CCCGGTTTGC CGCACTCCAG ATTGGGAAGG TGGCTGGGCT GCTGGCGTGC
MH142614.1  CCCGGTTTGC CGCACTCCAG ATTGGGAAGG TGGCTGGGCT GCTGGCGTGC
KX822142.1  CCCGGTTTGC CGCACTCCAG ATTGGGAAGG TGGCTGGGCT GCTGGCGTGC
KF486503.1  CCCGGTTTGC CGCACTCCAG ATTGGGAAGG TGGCTGGGCT GCTGGCGTGC
KF443217.1  CCCGGTTTGC CGCACTCCAG ATTGGGAAGG TGGCTGGGCT GCTGGCGTGC
KM506820.1  CCCGGTTTGC CGCACTCCAG ATTGGGAAGG TGGCTGGGCT GCTGGCGTGC
HM014280.1  CCCGGTTTGC CGCACTCCAG ATTGGGAAGG TGGCTGGGCT GCTGGCGTGC
DQ328751.1  CCCGGTTTGC CGCACTCCAG ATTGGGAAGG TGGCTGGGCT GCTGGCGTGC
KM506806.1  CCCGGTTTGC CGCACTCCAG ATTGGGAAGG TGGCTGGGCT GCTGGCGTGT
KM506805.1  CCCGGTTTGC CGCACTCCAG ATTGGGAAGG TGGCTGGGCT GCTGGCGTGT
KM506804.1  CCCGGTTTGC CGCACTCCAG ATTGGGAAGG TGGCTGGGCT GCTGGCGTGT
HM014269.1  CCCGGTTTGC CGCACTCCAG ATTGGGAAGG TGGCTGGGCT GCTGGCGTGC
HM014268.1  CCCGGTTTGC CGCACTCCAG ATTGGGAAGG TGGCTGGGCT GCTGGCGTGC

                      110        120        130        140        150
FmHe1       TGGTGGTGCA TTT-GCAGGT GGAGTGCGCC GAGGCGCCCG GTTCAGCGGC
FmHe2       TGGTGGTGCA TTT-GCAGGT GGAGTGCGCC GAGGCGCTCG GTTCAGCGGC
FmHe3       TGGTGGTGCA TTT-GCAGGT GGAGTGCGCC GAGGCGCCCG GTTCAGCGGC
AB933469.1  TGGTGGTGCA TTT-GCAGGT GGAGTGCGCC GAGGCGCTCG GTTCAGCGGC
MH156808.1  TGGTGGTGCA TTTTGCAGGT GGAGTGCGCC GAGGCGCCCG GTTCAGCGGC
MH142614.1  TGGTGGTGCA TTT-GCAGGT GGAGTGCGCC GAGGCGCCCG GTTCAGCGGC
KX822142.1  TGGTGGTGCA TTT-GCAGGT GGAGTGCGCC GAGGCGCCCG GTTCAGCGGC
KF486503.1  TGGTGGTGCA TTT-GCAGGT GGAGTGCGCC GAGGCGCCCG GTTCAGCGGC
KF443217.1  TGGTGGTGCA TTT-GCAGGT GGAGTGCGCC GAGGCGCCCG GTTCAGCGGC
KM506820.1  TTGTGGTGCA TTT-GCAGGT GGAGTGCGCT GAGGCGCTCG GCTCAGCGGC
HM014280.1  TTGTGGTGCA TTT-GCAGGT GGAGTGCGCT GAGGCGCTCG GCTCAGCGGC
DQ328751.1  TTGTGGTGCA TTT-GCAGGT GGAGTGCGCT GAGGCGCTCG GCTCAGCGGC
KM506806.1  CGGTGGTGCA TTT-GCAGGT GGAGTGCGCC GAGGCGCTCG GCTCAGCGGC
KM506805.1  CGGTGGTGCA TTT-GCAGGT GGAGTGCGCC GAGGCGCTCG GCTCAGCGGC
KM506804.1  CGGTGGTGCA TTT-GCAGGT GGAGTGCGCC GAGGCGCTCG GCTCAGCGGC
HM014269.1  TGGTGGTGCA TTT-GCAGGT GGAGTGCGCT GAGGCGCTCG GCTCGGCGGC
HM014268.1  TGGTGGTGCA TTT-GCAGGT GGAGTGCGCT GAGGCGCTCG GCTCGGCGGC

                      160        170        180        190        200
FmHe1       ATGAGCTCGG CTTTGAGGCC AGTCGCGCAA GCGGTCTGGT ACCCGGGTCG
FmHe2       ATGAGCTCGG CTTTGAGGCC AGTCGCGCAA GCGGTCTGGT ACCCGGGTCG
```

**FmHe3**	ATGAGCTCGG	CTTTGAGGCC	AGTCGCGCAA	GCGGTCTGGT	ACCCGGGTCG
**AB933469.1**	ATGAGCTCGG	CTTTGAGGCC	AGTCGCGCAA	GCGGTCTGGT	ACCCGGGTCG
**MH156808.1**	ATGAGCTCGG	CTTTGAGGCC	AGTCGCGCAA	GCGGTCTGTG	ACCCGGGTCG
**MH142614.1**	ATGAGCTCGG	CTTTGAGGCC	AGTCGCGCAA	GCGGTCTGGT	ACCCGGGTCG
**KX822142.1**	ATGAGCTCGG	CTTTGAGGCC	AGTCGCGCAA	GCGGTCTGGT	ACCCGGGTCG
**KF486503.1**	ATGAGCTCGG	CTTTGAGGCC	AGTCGCGCAA	GCGGTCTGGT	ACCCGGGTCG
**KF443217.1**	ATGAGCTCGG	CTTTGAGGCC	AGTCGCGCAA	GCGGTCTGGT	ACCCGGGTCG
**KM506820.1**	ATGAGCTCGG	CCTTGAGGCC	AACCGTGCAA	GCGGTCTGGT	ACCCGGGTCG
**HM014280.1**	ATGAGCTCGG	CCTTGAGGCC	AACCGTGCAA	GTGGTCTGGT	ACCCGGGTCG
**DQ328751.1**	ATGAGCTCGG	CCTTGAGGCC	AACCGTGCAA	GCGGTCTGGT	ACCCGGGTCG
**KM506806.1**	ATGAGCTCGG	CTTTGAGGCC	AACCGCGCAA	GCGGTCTGGT	ACCCGGGTCG
**KM506805.1**	ATGAGCTCGG	CTTTGAGGCC	AACCGCGCAA	GCGGTCTGGT	ACCCGGGTCG
**KM506804.1**	ATGAGCTCGG	CTTTGAGGCC	AACCGCGCAA	GCGGTCTGGT	ACCCGGGTCG
**HM014269.1**	ATGAGCTCGG	CTTTGAGGCC	AACCGCGCAA	GCGGTCTGGT	ACCCGGGTCG
**HM014268.1**	ATGAGCTCGG	CTTTGAGGCC	AACCGCGCAA	GCGGTCTGGT	ACCCGGGTCG

	210	220	230	240	250
**FmHe1**	GGGGAGTGTT	GTTTGGGCTG	GGTGTTCGC-	-TGGGGCACA	GGTTTCGGGC
**FmHe2**	GGGGAGTGTT	GTTTGGGCTG	GGTGTTCGC-	-TGGGGCACA	GGTTTCGGGC
**FmHe3**	GGGGAGTGTT	GTTTGGGCTG	GGTGTTCGC-	-TGGGGCACA	GGTTTCGGGC
**AB933469.1**	GGGGAGTGTT	GTTTGGGCTG	GGTGTTCGC-	-TGGGGCACA	GGTTTCGGGC
**MH156808.1**	GGGGAGTGTT	GTTTGGGCTG	GGTGTTCGC-	-TGGGGCACA	GGTTTCGGGC
**MH142614.1**	GGGGAGTGTT	GTTTGGGCTG	GGTGTTCGC-	-TGGGGCACA	GGTTTCGGGC
**KX822142.1**	GGGGAGTGTT	GTTTGGGCTG	GGTGTTCGC-	-TGGGGCACA	GGTTTCGGGC
**KF486503.1**	GGGGAGTGTT	GTTTGGGCTG	GGTGTTCGC-	-TGGGGTACA	GGTTTCGGGC
**KF443217.1**	GGGGAGTGTT	GTTTGGGCTG	GGTGTTCGC-	-TGGGGCACA	GGTTTCGGGC
**KM506820.1**	GGGGAGTGTT	GTTTGGGCTG	AGTGTTCGC-	-TTGGGCACT	GGTTTCGGGC
**HM014280.1**	GGGGAGTGTT	GTTTGGGCTG	AGTGTTCGC-	-TTGGGCACT	GGTTTCGGGC
**DQ328751.1**	GGGGAGTGTT	GTTTGGGCTG	AGTGTTCGC-	-TTGGGCACT	GGTTTCGGGC
**KM506806.1**	GGGGAGTGTT	GTTTGGGCTG	AGTGTTCGC-	-TTGGGCACT	GGTTTCGGGC
**KM506805.1**	GGGGAGTGTT	GTTTGGGCTG	AGTGTTCGC-	-TTGGGCACT	GGTTTCGGGC
**KM506804.1**	GGGGAGTGTT	GTTTGGGCTG	AGTGTTCGCG	CTTGGGCACT	GGTTTCGGGC
**HM014269.1**	GGGGAGTGTT	GTTCGGGCTG	AGTGTTCGC-	-TTGGGCACT	GGCATCGGGC
**HM014268.1**	GGGGAGTGTT	GTTCGGGCTG	AGTGTTCGC-	-TTGGGCACT	GGCATCGGGC

	260	270	280	290	300
**FmHe1**	CCGGGTGGGT	GCCGAGCTGG	CGGTCGGCGG	CGGTCGCGTG	CGACACGTGC
**FmHe2**	CCGGGTGGGT	GCCGAGCTGG	CGGTCGGCGG	CGGTCGCGTG	CGACACGTGC
**FmHe3**	CCGGGTGGGT	GCCGAGCTGG	CGGTCGGCGG	CGGTCGCGTG	CGACACGTGC
**AB933469.1**	CCGGGTGGGT	GCCGAGCTGG	CGGTCGGCGG	CGGTCGCGTG	CGACACGTGC
**MH156808.1**	CCGGGTGG-T	GCCGAGCTGG	CGGTCGGCGG	CGGTCGCGTG	CGACACGTGC
**MH142614.1**	CCGGGTGGGT	GCCGAGCTGG	CGGTCGGCGG	CGGTCGCGTG	CGACACGTGC
**KX822142.1**	CCGGGTGGGT	GCCGAGCTGG	CGGTCGGCGG	CGGTCGCGTG	CGACACGTGC
**KF486503.1**	CCGGGTGGGT	GCCGAGCTGG	CGGTCGGCGG	CGGTCGCGTG	CGACACGTGC
**KF443217.1**	CCGGGTGGGT	GCCGAGCTGG	CGGTCGGCGG	CGGTCGCGTG	CGACACGTGC
**KM506820.1**	CCGGGTGGGT	GCCGAGCTGG	CGGTCGACGT	CGGTCGCATG	CGACACGTGT
**HM014280.1**	CCGGGTGGGT	GCCGAGCTGG	CGGTCGACGT	CGGTCGCATG	CGACACGTGT
**DQ328751.1**	CCGGGTGGGT	GCCGAGCTGG	CGGTCGACGT	CGGTCGCATG	CGACACGTGT
**KM506806.1**	CCGGGTGGGT	GCCGAGCTGG	CGGTGGACGG	CGGTCGCGTG	CGACACGTGT
**KM506805.1**	CCGGGTGGGT	GCCGAGCTGG	CGGTGGACGG	CGGTCGCGTG	CGACACGTGT
**KM506804.1**	CCGGTTGGGT	GCCGAGCTGG	CGGTGGACGG	CGGTCGCGTG	CGACACGTGT
**HM014269.1**	CCGGGTGGGT	GCCGAGCTGG	CGGTCGACGG	CGGTCGCATG	CGGCACGTGT
**HM014268.1**	CCGGGTGGGT	GCCGAGCTGG	CGGTCGACGG	CGGTCGCATG	CGACACGTGT

	310	320	330	340	350
**FmHe1**	CAGCAGTCAG	TTCGGTCCTG	CTCGAGCTCT	CTCGTGTCCG	GCCTCGGTGT
**FmHe2**	CAGCAGTCAG	TTCGGTCCTG	CTCGAGCTCT	CTCGTGTCCG	GCCTCGGTGT
**FmHe3**	CAGCAGTCAG	TTCGGTCCTG	CTCGAGCTCT	CTCGTGTCCG	GCCTCGGTGT
**AB933469.1**	CAGCAGTCAG	TTCGGTCCTG	CTCGAGCTCT	CTCGTGTCCG	GCCTCGGTGT
**MH156808.1**	CAGCAGTCAG	TTCGGTCCTG	CTCGAGCTCT	CTCGTGTCCG	GCCTCGGTGT
**MH142614.1**	CAGCAGTCAG	TTCGGTCCTG	CTCGAGCTCT	CTCGTGTCCG	GCCTCGGTGT

KX822142.1 CAGCAGTCAG TTCGGTCCTG CTCGAGCTCT CTCGTGTCCG GCCTCGGTGT
KF486503.1 CAGCAGTCAG TTCGGTCCTG CTCGAGCTCT CTCGTGTCCG GCCTCGGTGT
KF443217.1 CAGCAGTCAG TTCGGTCCTG CTCGAGCTCT CTCGTGTCCG GCCTCGGTGT
KM506820.1 CAGCAATCAG TTCGGTCCTG CTCGAGCTCT CGCGTGCCCG GCCTCGGTGT
HM014280.1 CAGCAATCAG TTCGGTCCTG CTCGAGCTCT CGCGTGTCCG GCCTCGGTGT
DQ328751.1 CAGCAATCAG TTCGGTCCTG CTCGAGCTCT CGCGTGTCCG GCCTCGGTGT
KM506806.1 CTTCAGTCAG TTCGGTCCTG CTCGAGCTCT CGCGTGTCCG GCCTCGGTGT
KM506805.1 CTTCAGTCAG TTCGGTCCTG CTCGAGCTCT CGCGTGTCCG GCCTCGGTGT
KM506804.1 CTTCAGTCAG TTCGGTCCTG CTCGAGCTCT CGCGTGTCCG GCCTCGGTGT
HM014269.1 CAGCAATCAG TTCGGTCCTG CTCGAGCTCT CATGTGTCTG GCCTCGGTGT
HM014268.1 CAGCAATCAG TTCGGTCCTG CTCGAGCTCT CATGTGTCTG GCCTCGGTGT

360 370 380 390 400
FmHe1 AAAAGCCGGT CATCTGTCCG ACCCGTCTTG AAACACGGAC CAAGGAGTTT
FmHe2 AAAAGCCGGT CATCTGTCCG ACCCGTCTTG AAACACGGAC CAAGGAGTTT
FmHe3 AAAAGCCGGT CATCTGTCCG ACCCGTCTTG AAACACGGAC CAAGGAGTTT
AB933469.1 AAAAGCCGGT CATCTGTCCG ACCCGTCTTG AAACACGGAC CAAGGAGTTT
MH156808.1 AAAAGCCGGT CATCTGTCCG ACGGGTCTTG AAACACGGAC CAAGGAGTTT
MH142614.1 AAAAGCCGGT CATCTGTCCG ACCCGTCTTG AAACACGGAC CAAGGAGTTT
KX822142.1 AAAAGCCGGT CATCTGTCCG ACCCGTCTTG AAACACGGAC CAAGGAGTTT
KF486503.1 AAAAGCCGGT CATCTGTCCG ACCCGTCTTG AAACACGGAC CAAGGAGTTT
KF443217.1 AAAAGCCGGT CATCTGTCCG ACCCGTCTTG AAACACGGAC CAAGGAGTTT
KM506820.1 AAAAGCCGGT CATCTGTCCG ACCCGTCTTG AAACACGGAC CAAGGAGTTT
HM014280.1 AAAAGCCGGT CATCTGTCCG ACCCGTCTTG AAACACGGAC CAAGGAGTTT
DQ328751.1 AAAAGCCGGT CATCTGTCCG ACCCGTCTTG AAACACGGAC CAAGGAGTTT
KM506806.1 AAAAGCCGGT CATCTGTCCG ACCCGTCTTG AAACACGGAC CAAGGAGTTT
KM506805.1 AAAAGCCGGT CATCTGTCCG ACCCGTCTTG AAACACGGAC CAAGGAGTTT
KM506804.1 AAAAGCCGGT CGTCTGTCCG ACCCGTCTTG AAACACGGAC CAAGGAGTTT
HM014269.1 AAAAGCCGGT CATCTGTCCG ACCCGTCTTG AAACACGGAC CAAGGAGTTT
HM014268.1 AAAAGCCGGT CATCTGTCCG ACCCGTCTTG AAACACGGAC CAAGGAGTTT

410 420 430 440 450
FmHe1 AGCGTGTGCG CGAGTCATTG GGTGTTGAAA ACCCAGAGGC GTAATGAAAG
FmHe2 AGCGTGTGCG CGAGTCATTG GGTGTTGAAA ACCCAGAGGC GTAATGAAAG
FmHe3 AGCGTGTGCG CGAGTCATTG GGTGTTGAAA ACCCAGAGGC GTAATGAAAG
AB933469.1 AGCGTGTGCG CGAGTCATTG GGTGTTGAAA ACCCAGAGGC GTAATGAAAG
MH156808.1 AGCGTGTGCG CGAGTCATTG GGTGTTGAAA ACCCAGAGGC GTAATGAAAG
MH142614.1 AGCGTGTGCG CGAGTCATTG GGTGTTGAAA ACCCAGAGGC GTAATGAAAG
KX822142.1 AGCGTGTGCG CGAGTCATTG GGTGTTGAAA ACCCAGAGGC GTAATGAAAG
KF486503.1 AGCGTGTGCG CGAGTCATTG GGTGTTGAAA ACCCAGAGGC GTAATGAAAG
KF443217.1 AGCGTGTGCG CGAGTCATTG GGTGTTGAAA ACCCAGAGGC GTAATGAAAG
KM506820.1 AGCGTGTGCG CGAGTCATTG AGCGTTGAAA ACCCAAAGGC GCAATGAAAG
HM014280.1 AGCGTGTGCG CGAGTCATTG GGCGTTGAAA ACCCAAAGGC GCAATGAAAG
DQ328751.1 AGCGTGTGCG CGAGTCATTG GGCGTTGAAA ACCCAAAGGC GCAATGAAAG
KM506806.1 AGCGTGTGCG CGAGTCATTG GGCGTTGAAA ACCCAAAGGC GCAATGAAAG
KM506805.1 AGCGTGTGCG CGAGTCATTG GGCGTTGAAA ACCCAAAGGC GCAATGAAAG
KM506804.1 AGCGTGTGCG CGAGTCATTG GGCGTTGAAA ACCCAAAGGC GCAATGAAAG
HM014269.1 AGCGTGTGCG CGAGTCATTG GGCGTTGAAA ACCCAAAGGC GCAATGAAAG
HM014268.1 AGCGTGTGCG CGAGTCATTG GGCGTTGAAA ACCCAAAGGC GCAATGAAAG

460 470 480 490 500
FmHe1 TGAAGGTCTG CCTTGTGCAG CTGATGTGTG ATCCTGGACG CTGCGGCGTC
FmHe2 TGAAGGTCTG CCTTGTGCAG CTGATGTGTG ATCCTGGGCG CCGCGGCGCC
FmHe3 TGAAGGTCTG CCTTGTGCAG CTGATGTGTG ATCCTGGGCG CCGCGGTGCC
AB933469.1 TGAAGGTCTG CCTTGTGCAG CTGATGTGTG ATCCTGGGCG CCGCGGCGTC
MH156808.1 TGAAGGTCTG CCTTGTGCAG CTGATGTGTG ATCCTGGGCG CCGCGGCGCC
MH142614.1 TGAAGGTCTG CCTTGTGCAG CTGATGTGTG ATCCTGGGCG CCGCGGCGCC
KX822142.1 TGAAGGTCTG CCTTGTGCAG CTGATGTGTG ATCCTGGGCG CCGCGGCGCC
KF486503.1 TGAAGGTCTG CCTTGTGCAG CTGATGTGTG ATCCTGGACG CTGCGGCGTC
KF443217.1 TGAAGGTCTG CCTTGTGCAG CTGATGTGTG ATCCTGGACG CTGCGGCGTC
KM506820.1 TGAAGGTCTG CCTTGTGCAG CTGATGTGTG ATCCTGGGTG CTGCGGTGCC

```
HM014280.1    TGAAGGTCTG CCTTGTGCAG CTGATGTGTG ATCCTGGGTG CTGCGGTGCC
DQ328751.1    TGAAGGTCTG CCTTGTGCAG CTGATGTGTG ATCCTGGGTG CTGCGGTGCC
KM506806.1    TGAAGGTCTG CCTTGTGCAG CTGATGTGTG ATCCTGGGCG CTGCGGTGCC
KM506805.1    TGAAGGTCTG CCTTGTGCAG CTGATGTGTG ATCCTGGGCG CTGCGGTGCC
KM506804.1    TGAAGGTCTG CCTTGTGCAG CTGATGTGTG ATCCTGGGCG CTGCGGTGCC
HM014269.1    AGAGGGTCTG CCTTGTGCAG CTGATGTGTG ATCCTGGGCG CTGCGGTGCC
HM014268.1    TGAAGGTCTG CCTTGTGCAG CTGATGTGTG ATCCTGGGCG CTGCGGTGCC

                      510        520        530        540        550
FmHe1         CGGGCGCAAC ATAGTCCCGT CCCGACTGCA TGCAGTGGGG CGGAGACAGA
FmHe2         CGGGCGCAAC ATAGTCCCGT CCTGACTGCA TGCAGTGGGG CGGAGACAGA
FmHe3         CGGGCGCAAC ATAGTCCCGT CCCGACTGCA TGCAGTGGGG CGGAGACAGA
AB933469.1    CGGGCGCAAC ATAGTCCCGT CCCGACTGCA TGCAGTGGGG CGGAGACAGA
MH156808.1    CGGGCGCAAC ATAGTCCCGT CCCGAGAGCA TGCAGTGGGG CGGAGACAGA
MH142614.1    CGGGCGCAAC ATAGTCCCGT CCCGACTGCA TGCAGTGGGG CGGAGACAGA
KX822142.1    CGGGCGCAAC ATAGTCCCGT CCCGACTGCA TGCAGTGGGG CGGAGACAGA
KF486503.1    CGGGCGCAAC ATAGTCCCGT CCCGACTGCA TGCAGTGGGG CGGAGACAGA
KF443217.1    CGGGCGCAAC ATAGTCCCGT CCCGACTGCA TGCAGTGGGG CGGAGACAGA
KM506820.1    CGGGCGCAAC ATAGTCCCGT CCCGACTGCA TGCAGTGGGG CGGAGACAGA
HM014280.1    CGGGCGCAAC ATAGTCCCGT CCCGACTGCA TGCAGTGGGG CGGAGACAGA
DQ328751.1    CGGGCGCAAC ATAGTCCCGT CCCGACTGCA TGCAGTGGGG CGGAGACAGA
KM506806.1    CGGGCGCAAC ATAGTCCCGT CCCGACTGCA TGCAGTGGGG CGGAGACAGA
KM506805.1    CGGGCGCAAC ATAGTCCCGT CCCGACTGCA TGCAGTGGGG CGGAGACAGA
KM506804.1    CGGGCGCAAC ATAGTCCCGT CCCGACTGCA TGCAGTGGGG CGGAGACAGA
HM014269.1    CGGGCGCAAC ATAGTCCCGT CCCGACTGCA TGCAGAGGGG CGGAGACAGA
HM014268.1    CGGGCGCAAC ATAGTCCCGT CCCGACTGCA TGCAGAGGGG CGGAGACAGA

                      560
FmHe1         GCGTACGCGC TGAG
FmHe2         GCGTACGCGC TGAG
FmHe3         GCGTACGCGC TGAG
AB933469.1    GCGTACGCGC TGAG
MH156808.1    GCGTACGCGC TGAG
MH142614.1    GCGTACGCGC TGAG
KX822142.1    GCGTACGCGC TGAG
KF486503.1    GCGTACGCGC TGAG
KF443217.1    GCGTACGCGC TGAG
KM506820.1    GCGTACGCGC TGAG
HM014280.1    GCGTACGCGC TGAG
DQ328751.1    GCGTACGCGC TGAG
KM506806.1    GCGTACGCGC TGAG
KM506805.1    GCGTACGCGC TGAG
KM506804.1    GCGTACGCGC TGAG
HM014269.1    GCGTACGCGC TGAG
HM014268.1    GCGTACGCGC TGAG
```

Figure S1. Multiple sequence alignment of Fm *Helicotylenchus* (FmHe1, FmHe2 and FmHe3) and available close *Helicotylenchus* spp. (*H. dihystera* - AB933469.1, MH156808.1, MH142614.1, KX822142.1, KF486503.1, KF443217.1; *H. pseudorobustus* - KM506820.1, HM014280.1, DQ328751.1; *H. microlobus* - KM506806.1, KM506805.1, KM506804.1; *H. leiocephalus* - HM014269.1, HM014268.1) sequences of D2D3 expansion region of the 28S rDNA gene (560 bp).
